# Inflammatory cytokines and sleep parameters response to life style intervention in subjects with obese chronic insomnia syndrome

**DOI:** 10.4314/ahs.v21i3.31

**Published:** 2021-09

**Authors:** Fadwah M Al-Sharif, Shehab M Abd El-Kader

**Affiliations:** 1 Department of Medical Laboratory Technology, Faculty of Applied Medical Sciences, King Abdulaziz University, Jeddah, Saudi Arabia; 2 Department of Physical Therapy, Faculty of Applied Medical Sciences, King Abdulaziz University, Jeddah, Saudi Arabia

**Keywords:** Chronic primary insomnia, inflammatory cytokines, life style intervention, sleep quality

## Abstract

**Background:**

Chronic primary insomnia is a prevalent sleep disorder that is associated with adverse effects on health outcomes. Sleep disturbance is usually associated with abnormal level of systemic inflammation biomarkers.

**Objective:**

The aim of this study was to detect changes in sleep quality and inflammatory markers following weight loss among subjects with chronic primary insomnia.

**Material and methods:**

Eighty previously sedentary subjects with chronic primary insomnia subjects enrolled in this study, their age ranged from 32–51 year were randomly assigned to life style intervention group (group A, n=40) or control group (group B, n=40). Polysomnographic recordings for sleep quality assessment, IL-6, IL-10 and TNF-α were measured before and at the end of the study after six months.

**Results:**

There was a significant increase in the total sleep duration, sleep efficiency, sleep onset latency and IL-10 in addition to significant reduction in awake time after sleep onset, REM latency, IL-6 and TNF-α after 6 months of in group(A) as a result of weight loss program; while the results of the control group (group B) were not significant. Also, there were significant differences between both groups at the end of the study.

**Conclusion:**

Life style intervention modulates systemic inflammatory parameters and sleep quality among subjects with chronic primary insomnia.

## Introduction

The global prevalence of chronic insomnia symptoms is about 19% of people^1^,^2^. However, insomnia adversely affects general health as it is usually associated with increased rate of morbidly and mortality^3^,^4^. In addition, obesity is an important risk factor for poor sleep quality and insomnia^5^–^7^.

Sleep impairment leads to abnormal level of inflammatory cytokines and elevation in blood pressure 8,9. These abnormal changes may induces coronary artery disease^10^,^11^. However, low level of systemic inflammation may be an etiological factor in development of cancer^12^–^14^.

To the best of our knowledge, the present may be the first study for effect of life style intervention on sleep and systemic inflammation parameters among obese subjects with chronic insomnia syndrome. Therefore, the purpose of this investigation was to measure changes in inflammatory cytokines and sleep parameters following weight loss among subjects with chronic primary insomnia.

## Patients and methods

### Subjects

**Figure (1):**
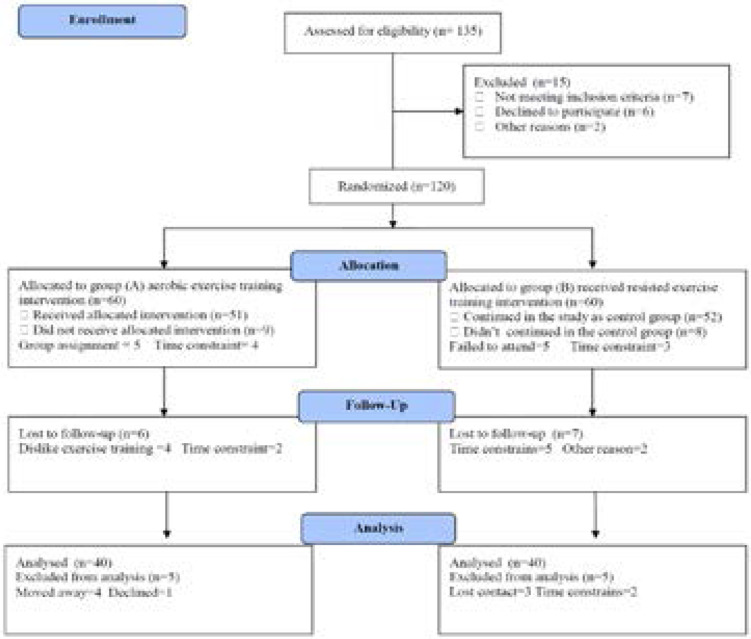
Subjects screening and recruitment CONSORT diagram.

Eighty previously sedentary subjects having Chronic Primary Insomnia for longer than six months, their age ranged from 32–51 years and participated in this study. Inclusion criteria included difficulty initiating sleep, difficulty maintaining sleep, early morning awakenings at least 3 nights during the previous 3 months. However, exclusion criteria included other sleep disorders include moderate or severe apnea, major depression, mental illness, cognitive impairment, cardiovascular disease, heart failure, liver disease, cancer, chronic pain conditions, shift work, regular use of sedatives, hypnotics and painkillers. The participated subjects were enrolled in two groups; group (A) received weight reducing program included training on treadmill and diet regimen. While, group (B) received no weight reducing program included training or treadmill and diet regimen and was the control group. The CONSORT diagram display the essential details of randomization (figure 1). Informed consent was obtained from all participants. This study was approved by the Scientific Research Ethical Committee, Faculty of Applied Medical Sciences at King University.

## Methods

### Measurements

A. Sleep measures: Recording of sleep parameters were measured by polysomnographic (PSG) (Philips-Respironics, USA) which was measured over 2 nights before the study and after six months at the end of the study 15.

B. Inflammatory cytokines: An overnight fasting venous blood samples were drained from the antecubital vein, the centrifuged blood samples were used to analyzed Interleukin-6 (IL-6) and Interleukin-10 (IL-10) levels with “Immulite 2000” immunassay analyzer (Siemens Healthcare Diagnostics, Deerfield, USA). While, ELISA microplate reader (ELX 808; BioTek Instruments, USA) was used to measure tumor necrosis factor-alpha (TNF-α).

### Procedures

Participants were enrolled randomly into the following groups:

1. Group (A): received weight reducing program included training on treadmill and diet regimen. The aerobic exercise was conducted on treadmill (Enraf Nonium, Model display panel Standard, NR 1475.801, Holland), The training program composed of warming-up for 5 minutes, aerobic exercise for 30 minutes of 60%–70% of maximal heart rate in addition to cooling down for 1o minutes, three sessions/ week for six months. While, the diet control provided 1200 Kilocalories/day for six months.

2. Group (B): received no weight reducing program included training or treadmill and diet regimen and was the control group.

### Statistical analysis

All the data were collected and entered in IBM SPSS version 21 and analyzed through its statistical package. All the variables were worked out and results were analyzed and presented in tables. The paired “t” test was used to compare the mean values of the investigated parameters in both groups. While Independent “t” test was used for comparison between the two groups (P<0.05).

## Results

The two groups were considered homogeneous regarding the demographic variables (table 1). The mean age of group, (A) was 46.12 ± 3.65 year, and the mean age of group (B) was 44.87 ± 4.32 year. There was no significant differences in age, gender, body mass index (BMI), body fat, systolic blood pressure, diastolic blood pressure, hemoglobin and maximal heart rate (HRmax) between both groups.

**Table 1:** Baseline characteristics of study participants

Characteristic	Group (A)	Group (B)	Significance
**Age** (years)	45.15 ± 5.27	43.76 ± 6.12	P>0.05
**Gender** (male/female)	23/17	22/18	P>0.05
**BMI** (kg/m^2^)	32.25 ± 4.14	32.17 ± 4.29	P>0.05
**Waist hip ratio**	0.89 ± 0.28	0.87 ± 0.21	P>0.05
**SBP** (mmHg)	135.61 ± 6.27	132.43 ± 7.11	P>0.05
**DBP** (mmHg)	84.29 ± 4.31	83.12 ± 4.17	P>0.05
**Hb** (gm/dl)	11.58 ± 1.59	12.13 ± 1.36	P>0.05
**HR_max_** (beat/min)	159.72 ± 11.61	160.35 ± 10.49	P>0.05

BMI: Body mass index; SBP: Systolic blood pressure; DBP: Diastolic blood pressure; Hb: Hemoglobin; HR_max_: Maximum heart rate.

There was a significant reduction in BMI, CD3, CD4 and CD8 awake time after sleep onset, REM latency, IL-6 and TNF-α in addition to significant increase in the total sleep duration, sleep efficiency, sleep onset latency and IL-10 after 6 months of in group(A) as a result of weight loss program (table 2); while the results of the control group (group B) were not significant (table 3). Also, there were significant differences between both groups at the end of the study(table 4).

**Table (2):** Mean value and significance of sleep parameters and inflammatory markers of group (A) before and at the end of the study

	Mean + SD		t-value	Significance
	Pre	Post		
**BMI** (kg/m^2^)	32.25 ± 4.14	27.93 ± 3.11*	8.36	P<0.05
**Total sleep** **duration** (min)	316.73 ± 28.18	348.45 ± 21.39*	12.51	P <0.05
**Sleep efficiency** (%)	65.91 ± 5.14	81.26 ± 6.25*	9.22	P <0.05
**Sleep onset** **latency** (min)	11.12 ± 3.43	15.28 ± 3.87*	7.19	P <0.05
**Awake time after sleep** **onset** (min)	78.23 ± 6.74	62.54 ± 5.12*	9.14	P <0.05
**REM sleep latency** (min)	88.42 ± 7.15	69.31 ± 6.84*	9.47	P <0.05
**TNF-α** (pg/mL)	5.24 ± 1.65	3.26 ± 1.37*	6.28	P <0.05
**IL-6** (pg/mL)	2.83 ± 0.94	1.71 ± 0.86*	5.64	P <0.05
**IL-10** (pg/ml)	5.72 ± 1.46	7.98 ± 1.63*	6.11	P <0.05

BMI: Body mass index; REM: rapid eye movements; TNF-α: tumor necrosis factor – alpha; IL-6: Interleukin-6; IL-10: Interleukin-10

* indicates a significant difference between the two groups, P < 0.05.

**Table (3):** Mean value and significance of sleep parameters and inflammatory markers of group (B) before and at the end of the study

	Mean + SD		t-value	Significance
	Pre	Post		
**BMI** (kg/m^2^)	32.17 ± 4.29	32.98 ± 4.31	0.543	P>0.05
**Total sleep** **duration** (min)	319.26 ± 26.95	322.75 ± 27.13	0.682	P>0.05
**Sleep efficiency** (%)	66.78 ± 6.15	64.17 ± 6.11	1.24	P>0.05
**Sleep onset latency** (min)	11.52 ± 2.61	10.95 ± 2.42	0.96	P>0.05
**Awake time after sleep** **onset** (min)	77.82 ± 7.19	79.14 ± 7.35	1.37	P>0.05
**REM sleep latency** (min)	90.17 ± 8.26	91.85 ± 8.31	1.24	P>0.05
**TNF-α** (pg/mL)	4.92 ± 1.75	5.11 ± 1.79	0.541	P>0.05
**IL-6** (pg/mL)	2.73 ± 0.78	2.91 ± 0.82	0.432	P>0.05
**IL-10** (pg/ml)	5.98 ± 1.36	5.65 ± 1.29	0.597	P>0.05

BMI: Body mass index; REM: rapid eye movements; TNF-α: tumor necrosis factor – alpha; IL-6: Interleukin-6; IL-10: Interleukin-10.

**Table (4):** Mean value and significance of sleep parameters inflammatory markers in group (A) and group (B) at the end of the study

	Mean + SD		t-value	Significance
	Group (A)	Group (B)		
**BMI** (kg/m^2^)	27.93 ± 3.11*	32.98 ± 4.31	7.25	P<0.05
**Total sleep** **duration** (min)	348.45 ± 21.39*	322.75 ± 27.13	11.14	P <0.05
**Sleep efficiency** (%)	81.26 ± 6.25*	64.17 ± 6.11	7.43	P <0.05
**Sleep onset** **latency** (min)	15.28 ± 3.87*	10.95 ± 2.42	6.38	P <0.05
**Awake time after sleep** **onset** (min)	62.54 ± 5.12*	79.14 ± 7.35	7.29	P <0.05
**REM sleep** **latency** (min)	69.31 ± 6.84*	91.85 ± 8.31	8.17	P <0.05
**TNF-α** (pg/mL)	3.26 ± 1.37*	5.11 ± 1.79	5.21	P<0.05
**IL-6** (pg/mL)	1.71 ± 0.86*	2.91 ± 0.82	4.13	P<0.05
**IL-10** (pg/ml)	7.98 ± 1.63*	5.65 ± 1.29	5.28	P<0.05

BMI: Body mass index; REM: rapid eye movements; TNF-α: tumor necrosis factor – alpha; IL-6: Interleukin-6; IL-10: Interleukin-10

* indicates a significant difference between the two groups, P < 0.05.

## Discussion

Sleep loss alters molecular processes that induce low grade of systemic inflammation^16^ as well as increased circulating levels of inflammatory markers (i.e., IL-6, tumor necrosis factor-alpha TNF-α, C-reactive protein) 17,18. Therefore, the aim of this study was to measure chages in inflammatory cytokines and sleep parameters following weight loss among subjects with chronic primary insomnia.

Concerning sleep quality parameter, the present study found significant improvement in all sleep parameters as a result of weight loss in group(A), these results are in line with Tan et al. reported that modest energy restriction as a result of six-month individualized diet intervention shorten sleep onset latency in overweight and obese men with insomnia symptoms 19. In addition, Tan and colleagues reported that a six months weight reducing program significantly improved sleep quality parameters in obese men enrolled 20. However, Passos et al. stated that a four-months aerobic exercise training program resulted in improvement in sleep parameters among subjects with chronic primary insomnia 21. Many mechanisms may be responsible for improvement in sleep parameters as a result of weight reduction included changes in composition of gut microbiota and specific hormones in addition to anti-inflammatory cytokines and endorphin secretion 22–24.

To our knowledge, this is limited number of researches that measure inflammatory cytokines response to intentional weight loss in subjects with chronic primary insomnia. Our results indicate that intentional weight loss modulated systemic inflammation markers. These results are in line with several previous studies reported that CRP and TNF-α are known to decrease by weight loss^25^–^27^. Many previous studies proved that weight reducing program was an effective treatment modality in obese subjects with OSA 28–30. However, lifestyle modification modulated inflammatory cytokines in diabetic patients 31. Moreover, Cotie et al. proved that 4-months of exercise training and diet regimen improved inflammatory markers as a result of weight reduction among obese women 32. However, Lang et al. reported that two months weight control resulted in modulation of blood lipids and inflammatory markers of obese subjects 33. While, Madsen et al. stated that inflammatory cytokines significantly improved as a result of weight loss in obese individuals 34. Sheu et al. enrolled 21 non-diabetic obese women in a three months weight reducing program which resulted in significant reduction in TNF-α and IL-6 35. Finally, Sahlman et al. enrolled overweight patients with mild OSA in a one year supervised lifestyle intervention which resulted in reduction of inflammatory cytokines 36. The possible mechanism of inflammatory cytokines modulation as result of weight reducing program may be due to reduced fat mass 37–40.

## Conclusion

Life style intervention modulates systemic inflammation and sleep parameters among obese subjects with Chronic Primary Insomnia.
